# Evaluation of the SD FK70 Malaria Ag *Plasmodium vivax *rapid diagnostic test in a non-endemic setting

**DOI:** 10.1186/1475-2875-8-129

**Published:** 2009-06-11

**Authors:** Philippe Gillet, Katrien Bosselaers, Lieselotte Cnops, Emmanuel Bottieau, Marjan Van Esbroeck, Jan Jacobs

**Affiliations:** 1 Department of Clinical Sciences, Institute of Tropical Medicine (ITM), Antwerp, Belgium; 2 Department of Medical Microbiology, Faculty of Health, Medicine and Life Sciences (FHML), Maastricht, the Netherlands

## Abstract

**Background:**

For clinical and epidemiological reasons, it is interesting to diagnose non-*falciparum *malaria to the species level. This retrospective study assessed the performance of the SD BIOLINE Malaria Antigen Pv test (FK70), a two-band immunochromatographic test detecting *Plasmodium vivax*-specific lactate dehydrogenase, on samples of international travellers in a non-endemic setting.

**Methods:**

Stored blood samples from international travellers suspected of malaria were used, with microscopy corrected by PCR as the reference method. Samples infected by *Plasmodium vivax *(n = 100), *Plasmodium falciparum *(n = 75), *Plasmodium ovale *(n = 75) and *Plasmodium malariae *(n = 25) were included, as well as 100 malaria-negative samples. End points were sensitivity, specificity, inter-reader reliability and reproducibility.

**Results:**

The overall sensitivity of the FK70 for the diagnosis of *P. vivax *was 88.0% (95% confidence interval (CI): 83.6% – 90.3%). For parasite densities > 500/μl, a sensitivity of 97.2% (CI: 92.6% – 99.1%) was obtained. Specificity was 98.5%, with 4 out of 75 *P. falciparum *samples testing positive. None of the *P. ovale *samples tested positive. Nearly two-thirds (57/88, 64.7%) of positive *P. vivax *samples showed faint or weak line intensities, with stronger line intensities at higher parasite densities. The test showed excellent reproducibility and reliability for test results and line intensities (kappa values exceeding 0.98 and 0.87 respectively).

**Conclusion:**

The FK70 test performed well in diagnosing *P. vivax *infections in a non-endemic reference setting. It can be of added value to microscopy in species differentiation of malaria infections, especially at parasite densities > 500/μl.

## Background

Malaria is a condition that requires prompt diagnosis, for which microscopy is considered as the reference standard. Although cheap, reliable and available on an instant base, microscopy has its limitations. For instance, in resource-limited centres, there are problems of equipment, training and workload, whereas in non-endemic countries, laboratory staff may lack sufficient exposure to malaria positive samples resulting in low expertise [1,2]. Malaria rapid diagnostic tests (RDTs) have proven to be a valuable adjunct to microscopy. The tests initially developed included a test control line and a *Plasmodium falciparum*-specific line targeting histidine-rich protein-2 (HRP-2) or *P. falciparum*-specific parasite lactate dehydrogenase (pLDH). These so-called two-band tests only detect *P. falciparum*. The three-band test, engineered later, comprises a third line for detection of an antigen common to the four *Plasmodium *species, such as pan *Plasmodium*-specific pLDH or aldolase. These RDTs still only distinguish between infections due to *P. falciparum *(or a mix of *P. falciparum *and one or more of the non-*falciparum *species) on the one hand, and infections with any of the non-*falciparum *species on the other hand. Although monoclonal antibodies directed to *Plasmodium vivax*-specific pLDH (Pv-pLDH) have been developed, RDTs addressing this target have undergone only limited evaluation [3,4].

The SD FK70 Malaria Antigen Pv test (05-FK70-02-0, Standard Diagnostics, Hagal-Dong, Republic of Korea), further referred to as FK70, is a two-band RDT that targets *P. vivax*-specific pLDH. This test was evaluated as part of an accreditation process in the laboratory.

## Methods

### Study design

In this retrospective study, the FK70 was evaluated against a collection of stored samples obtained from international travellers. Tests were carried out in the reference laboratory of the Institute of Tropical Medicine (ITM) Antwerp, Belgium.

### Patients and samples

Samples were selected from a collection of EDTA-blood samples stored at -70°C and obtained from patients presenting at the outpatient clinic of ITM. The patients were international travellers and, to a lesser extent, immigrants returning from visits to their native countries. In addition, samples sent by Belgian laboratories to ITM in the scope of the national reference function were included. A representative number of samples were selected (n = 375), including the four malaria species with varying parasite densities and representative geographic distribution.

### Reference method

All samples were analysed by microscopy and real-time polymerase chain reaction (PCR). In case of discordant results, microscopy was corrected by PCR as the reference method. Standard microscopy was performed on thick blood films of all samples to diagnose malaria and to assess parasite density, and on thin blood films of positive samples to define the *Plasmodium *species. Thick blood films were stained with Giemsa 3.5% (Merck, KGmA, Darmstadt, Germany) (pH = 8.0) for 20 minutes, thin films with May-Grünwald Giemsa. The slides were examined by light microscopy using a × 500 magnification, according to the standard procedure at ITM. Parasite density was assessed by counting the number of asexual parasites against 200 white blood cells in a thick film, converting this to parasites/μl using the actual count or the standard of 8,000 white blood cell/μl [2].

Real-time PCR analysis was adapted from Rougemont *et al *[5] and had been validated against as part of ISO15189 accreditation. DNA of all 375 samples was extracted with a QIAamp DNA minikit (Qiagen Benelux, Venlo, the Netherlands) using the same set of primers and species-specific probes (Biolegio, Nijmegen, the Netherlands), with reporters and quenchers adapted to the dye channels of the Cepheid Smart Cycler II device (Lucron Bioproducts, De Pinte, Belgium). Two PCR reactions were run in parallel, a duplex reaction to detect *P. falciparum *and *P. vivax *and another reaction for *P. ovale *and *P. malariae*. In the first reaction, the 25 μl reaction mix contained 5 μl DNA, 1× Quantitec mix (Qiagen Benelux), 200 nM forward and reverse primer, 100 nM *falciparum *probe and 200 nM *vivax *probe. In the second reaction, besides 5 μl template DNA and 1× Quantitec mix (Qiagen Benelux), 500 nM forward and reverse primer, 320 nM *ovale *probe, 200 nM *malariae *probe and 1 mM MgCl_2 _were added. The PCR programme consisted of an initial step of 15 min at 95°C followed by 50 cycles of 5 sec at 95°C, 30 sec at 58°C and finally 30 sec at 72°C.

### Test platform

The FK70 is a lateral flow immunochromatographic RDT in a cassette format. Two lines are present, a control line, which indicates whether the test is valid, and a Pv-pLDH line. According to the manufacturer's instructions, any visible Pv-pLDH line should be considered as positive, and results are expressed as positive or negative for *P. vivax*.

For the evaluation, test kits of two different lot numbers were used, RDT7001 and BD7001 with expiry dates of 12.08.09 and 27.08.09 respectively.

### Test procedure

Tests were performed according to the instructions of the manufacturer, except that samples (5 μl) were loaded with a transfer pipette (Finnpippette, Helsinki, Finland) instead of the plastic loop supplied by the manufacturer and that a scoring system was used to assess the intensity of the Pv-pLDH line. In case the control line did not appear, the result was interpreted as invalid and the test was repeated. In order to score Pv-pLDH line intensities, the scoring system of Bell and co-workers [6] was applied and defined five categories: none (no line visible), faint (barely visible line), weak (paler than the control line), medium (equal to the control line) or strong (stronger than the control line). To assure timely readings, tests were carried out in time-controlled batches of ten samples. Readings were performed by three subsequent readers, of whom the one who performed the test procedure invariably was the first. Readers were blinded to the results of microscopy and to each others' readings. Readings were carried out at daylight assisted by a standard electricity bulb, between 20 and 30 minutes (but not beyond) after application of the sample and buffer. The results of the readings considered were based on consensus agreement, which means that the same result was observed by at least two out of three different readers. When there was no consensus, results of the first reader were considered. Inter-reader reliabilities were assessed for the test results expressed as positive and negative readings as well as for the Pv-pLDH line intensity readings. To assess reproducibility, a panel of 25 samples (including 20 *P. vivax *samples, four *P. falciparum *samples and one *P. ovale *sample) was tested on three successive occasions.

### Statistical analysis

True positive results were defined as those with a Pv-pLDH line visible in samples with *P. vivax *seen at microscopy, and true negative results as those with no Pv-pLDH line visible in microscopy-negative samples and in samples with other *Plasmodium *species. False-negative samples were identified as those with a microscopic diagnosis of *P. vivax*, but no Pv-pLDH line visible, and false-positive samples as microscopic negative samples and samples with non-*vivax Plasmodium *showing a Pv-pLDH line.

From these categories sensitivity and specificity were calculated with 95% confidence intervals (C.I.). Reliabilities for positive and negative readings and line intensities were calculated as percentage agreements for all three readers and kappa values for each pair of readers. Differences between proportions were tested for significance using the chi-square test or, in case of small sample sizes, a two-tailed Fisher's exact test. A p-value < 0.05 was considered as significant. Associations between line intensity readings and parasite densities were assessed for strength of association with Cramer's *V *for categorical variables.

### Ease of use

Three experienced laboratory technicians scored the ease of use of the FK70 test and the clarity of manufacturer's instructions with a standardized list.

### Ethical review

The study was reviewed and approved by the Institutional Review Board of ITM and by the Ethical Committee of Antwerp University, Belgium.

## Results

### Sample collection

Out of 1,324 stored samples of ITM, 375 samples were selected, of which 49 samples were sent by Belgian laboratories to ITM for second opinion. The samples were collected from December 1995 to November 2007. According to microscopy and after correction for PCR analysis, 100 of these samples were positive for *P. vivax*, 75 for *P. ovale*, 75 for *P. falciparum *and 25 for *P. malariae*. The results of microscopy were corrected in 11 out of 375 (2.9%) samples and were uniquely related to *P. vivax *– *P. ovale *mismatches: 4 out of 75 (5.3%) and 7 out of 100 (7.0%) samples that had been categorised as *P. vivax *and *P. ovale *by the original microscopy were identified as *P. ovale *and *P. vivax *respectively by PCR. The microscopic identification of all *P. falciparum*, and *P. malariae *samples as well as the results of microscopy-negative samples were confirmed by PCR.

In addition, 100 microscopic and PCR negative samples of symptomatic travellers were included in the panel. The majority of the *P. vivax *samples had been acquired in Asia.

### Invalid test results

Two of the 375 samples gave invalid results at initial testing. After application into the respective well, neither the blood nor the buffer started to migrate. Upon repetition, tests performed well.

### Sensitivity and specificity

Table 1 lists test characteristics matched with species identification and parasite density. The sensitivity at parasite densities > 500/μl was 97.2% (Confidence Interval (CI): 92.6% – 99.1%) but was less at lower parasite densities < 500/μl (64.3%, p < 0.001), with 10 out of 12 false negative samples occurring in this category. Challenged with other *Plasmodium *species and negative samples, the FK70 showed a specificity of 98.5%, with 4 out of 75 (5.3%) *P. falciparum *samples showing cross-reactions. Of interest is that none out of 75 *P. ovale *samples tested positive.

**Table 1:** Test characteristics of the FK70

**Species and parasite density (asexual parasites/μl)**	**Numbers tested**	**Numbers tested positive by FK70**	**Sensitivity % (95% CI)**	**Specificity % (95% CI)**
*P. vivax*, 0–500/μl	28	18	64.3 (51.0 – 72.3)	

*P. vivax*, 501–1,000/μl	11	11	100.0 (78.8 – 100.0)	

*P. vivax*, > 1,000/μl	61	59	96.7 (91.3 – 99.0)	

*P. vivax*, all samples combined	100	88	88.0 (83.6 – 90.3)	

Other *Plasmodium *species	175	4		98.5 (96.9 – 99.4)

*P. falciparum*	75	4		

*P. ovale*	75	0		

*P. malariae*	25	0		

Malaria negative samples	100	0		

Test characteristics of the FK70 matched with species and parasite density, PCR-corrected microscopy as the reference method (n = 375).

### Line intensities

Table 2 lists the Pv-pLDH line intensities matched to species identification and parasite density. Nearly two-thirds (57/88, 64.7%) of all positive *P. vivax *samples generated faint or weak line intensities. These occurred not only at low parasite densities (< 500/μl), but also in 8/11 samples and 31/61 samples of parasite densities between 501–1,000/μl and > 1,000/μl respectively. Line intensity readings were significantly related to parasite densities with a substantial correlation (Cramer's *V *= 0.5633, p < 0.001), but there was considerable overlap between categories.

**Table 2:** Line intensities of the Pv-PLDH line

	Consensus readings of Pv-pLDH line*					
**Species and parasite density**	**Negative**	**Faint**	**Weak**	**Medium**	**Strong**	**Total**

*P. vivax *0–500/μl	10	4	14			28

*P. vivax *501–1,000/μl		1	7	2	1	11

*P. vivax *> 1,000/μl	2	4	27	17	11	61

*P. falciparum*	71		4			75

*P. ovale*	75					75

*P. malariae*	25					25

Malaria negative samples	100					100

**Total**	**283**	**9**	**52**	**19**	**12**	**375**

FK70 line intensity of the Pv-pLDH line for the different parasite densities

* Consensus Readings: line intensity as read by at least two out of three readers

### Inter-reader reliability

Inter-reader reliability for positive and negative test results was high, with 99.2% agreement between the three readers and kappa values exceeding 0.98 for each pair of readers. Two samples of *P. vivax *with parasite densities of 82 and 230/μl were reported as negative by one reader while the other two readers reported them as faint; an additional sample with a parasite density of 10,556/μl was reported as faint by two readers and as negative by the third one. Reliability for Pv-pLDH line intensity readings was also high, with an overall agreement of 94.2% and kappa values between the different pairs of readers of 0.87, 0.88 and 0.92 respectively. For strong intensities, all readings were identical. Less consistent readings were obtained for other intensities, but discordances between two readers were always within one category of difference.

### Reproducibility

Test results and Pv-pLDH line intensity readings were reproducible. Consistent line intensity readings for all readers upon three times repetition were obtained for 12 of the 25 samples. For 10 other samples, discordances occurred only within one category of difference in line intensity. The three remaining samples showed differences in more than one category of difference, with negative readings, faint and/or weak readings.

### Analysis of discordant results

For sixteen samples, the FK70 and PCR-corrected microscopy provided discordant results. Twelve *P. vivax *samples tested negative and four *P. falciparum *samples tested positive. The false-negative *P. vivax *samples showed no particular geographic distribution and most of them had low parasite densities (< 500/μl) (Table 2). The four false positive *P. falciparum *samples showed weak line intensities; they were acquired in the Cameroon, Guinea, the Democratic Republic of the Congo, and Nigeria (the latter two in autochthonous native Africans) and had parasite densities of 7,000, 158,000, 264,000 and 1,000,000/μl, respectively. All samples gave identical results upon retesting except for a single *P. falciparum *sample (with parasite density of 7,000/μl) that was negative upon retesting. Real-time PCR for all four samples was conclusive for a single species *P. falciparum *infection. The samples represented 3/16 samples with parasite densities above 100,000/μl and 1/59 samples with parasite densities lower than 100,000/μl (p < 0.05).

### Ease of use

The FK70 was scored as practical and easy in use and the instructions were scored as clear and simple to perform by all three technicians.

## Discussion

This retrospective study demonstrated that the two-band FK70 test performed well for the detection of *P. vivax *in a non-endemic reference setting. The FK70 was scored as easy-to-use and showed an overall sensitivity and specificity of 88.0% and 98.5% respectively, with excellent reliability and reproducibility.

Several limitations of this study have to be considered. Its retrospective design precluded exploration of discrepant results, and although the representative malaria negative control samples (i.e. samples of symptomatic international travellers), the test was not challenged with samples that are known to cause false positive results such as those presenting the rheumatoid factor [7]. Next, calibrated transfer pipette was used instead of the manufacturer's transfer device, thereby bypassing a possible error of the kit's application loop. An effect of storage of the samples on the test results cannot be excluded, but seems to be unlikely, as observed similar patterns of test results and Pv-pLDH line intensities on fresh samples analysed prospectively after introduction of the FK70 in the ITM malaria reference laboratory. Finally, it should be noted that the present findings apply to a non-endemic reference setting and should not be as such extrapolated to field settings: for instance, reported sensitivities of RDTs tend to be lower in field settings as compared to reference settings due to better test conditions in the latter [3,8]. Likewise, trained lab technicians generally tend to score tests and instructions more favourably than less trained end-users in remote settings, who may experience more practical difficulties when handling the RDTs [9].

It is difficult to compare the present findings to the reported literature. Although RDTs have been extensively evaluated for the diagnosis of *P. falciparum *malaria, their ability to detect the other *Plasmodium *species is less documented. Most studies have investigated small numbers of the non-*falciparum *species, resulting in low precisions and explaining in part for the wide range in reported sensitivities [3,10]. Adding to the difficulties are the evolutions in proprietary compositions and brand names of RDTs, with continuous releases of improved designs as well as well-studied products withdrawn from the market [11]. The World Health Organization (WHO) lists RDT manufacturers and distributors online [12], but it is not clear from the list whether in case a particular RDT detects *P. vivax *by Pv-pLDH or by pan-*Plasmodium*-pLDH. According to the literature, the DBEST One Step Malaria Test (AmeriTek, Everett, WA, USA) and the FK70 are at present the only marketed two-band RDTs that specifically detect *P. vivax*. In general, sensitivities of three-band RDTs for the detection of *P. vivax *tend to be somewhat lower than presently described for the two-band FK70. As an example, compiled sensitivity for the BinaxNOW kit has been calculated as 69.6% [3], and reported sensitivities for other kits range from 1.5% to 97% [3,13]. Decreasing sensitivities at parasite densities below 5,000/μl and particularly below 500/μl are reported for all kits [4,14].

Two previous studies assessed the performance of the FK70 in a *P. vivax*-endemic setting. In a prospective clinical study, Lee and co-workers demonstrated sensitivity and specificity of 96.4% and 98.9% respectively [15]. However, they did not include data on parasite densities and the control group only consisted of malaria-negative samples. Likewise, Kim and co-workers found in a prospective study an overall sensitivity of 93.4% with lower sensitivities at low parasite densities, albeit somewhat higher as compared to those found in the present study. Among asymptomatic patients and international travellers with *P. falciparum *infections, they found no false-positive reactions. Compared side-by-side, their results for the FK70 were in line with those obtained with the three-band OptiMAL test (Diamed, Cressier, Switzerland), which detects *P. vivax *through a pan-Plasmodium-pLDH antibody [16]. In contrast to both studies, the FK70 was challenged with a large number of non-*vivax *species, reflecting more appropriately a travel clinic setting. Unlike the study of Kim and co-workers, this study demonstrated cross-reactions with the Pv-pLDH line in four *P. falciparum *samples: these samples were obtained in travellers returning from Africa, and co-infection with *P. vivax *was not demonstrated by PCR-analysis. As the presently used PCR analysis had been validated with large numbers of the four main *Plasmodium *species that mixed infection with *P. vivax *is extremely unlikely and an immunological cross-reaction has to be concluded. Of note is that the three samples in which this cross-reaction was found reproducible had parasite densities exceeding 100,000/μl.

The Pv-pLDH line intensities in the present study were reproducible and reliable but most readings were scored as faint or weak. As noted above, this might be caused by a decrement of antigen activity during storage [3], but this phenomenon was also demonstrated in recently stored samples is also noted in prospectively assessed samples at the ITM reference laboratory. Further studies are to be done in order to confirm and document this observation. Faint test lines are a frequently described problem, which may be caused by the fact that the test line is much narrower than the control line [17,18]. The phenomenon is important especially in field conditions: interpretation of faint positive lines as negative test results was found the most common mistake made by community health care workers even when provided with adapted job aids [19,20] and problems can be expected when reading RDTs during evening and night shifts, particularly for readers with poor visual capacities [21].

In our hands, the FK70 proved to be an easy-to-use test. The package insert was scored as clear and informative. The additional job aids is based on the firstly developed WHO generic version of the job aids [22] with easily visible and readable instructions.

Which is the place of a RDT targeting uniquely *P. vivax*? Malaria caused by *P. vivax *is no longer considered as a mild infection, and compiled evidence has demonstrated complicated infections in endemic countries as well as in returned travellers [23,24]. *Plasmodium vivax *is further notorious because of its high transmissibility, which makes it more difficult to eradicate as compared to *P. falciparum *[25]. It has a tendency to relapse (for which primaquine therapy is needed), and chloroquine resistant strains are reported from various parts in the world [26-28]. As to its epidemiology and geography, it should be noted that *P. vivax *unexpectedly has been recovered from travellers returning from Central-Africa, where it is however rarely found in indigenous populations, due to their lack of the Duffy antigen [29]. In the non-endemic setting, the microscopic differentiation between *P. vivax *and *P. ovale *is notoriously difficult [4], with 14 tot 42% laboratories participating to external quality assessments confusing the one species with the other [30]. The rare and even decreasing trends of *P. vivax *and *P. ovale *infections in returned travellers contribute to the difficulty of correct species identification [31]. In view of these difficulties, kits such as the FK70 may be a useful aid for the instant differential diagnosis of *Plasmodium *species. The affordable price, long shelf life and favourable storage conditions of the kit allow purchase for incidental use. At present, the FK70 is used in our laboratory as part of the work-up of samples microscopically suspected for *P. vivax *and/or *P. ovale*. In areas where *P. vivax *is endemic and occurs as a single infection, a *P. vivax *specific RDT can be of value because it is – at an better accuracy – cheaper than a three-band RDT [32,33]. In areas where *P. vivax *and *P. falciparum *are co-circulating, it can distinguish those infections that require treatment with primaquine [3]. However, in view of the potential cross-reaction of *P. falciparum*, the FK70 should not be used the initial or unique screening test in this situation as an occasional concurrent or single *P. falciparum *infection risks to be overlooked.

## Conclusion

In conclusion, the FK70 proved to be easy-to-use and accurate and reliable for the diagnosis of *P. vivax *among a panel of samples of symptomatic international travellers. It is a useful adjunct to microscopy in non-endemic settings. Lower sensitivities at parasite densities below 500/μl and occasional cross-reactions of *P. falciparum *should be taken into account and further prospective investigations should be done to explore the Pv-pLDH line intensities, especially when considering the use of the FK70 in a field setting.

## Abbreviations

CI: confidence intervals; EDTA: ethylene diamine Tetra-acetic Acid; FK70: SD FK70 Malaria Antigen Pv test; HRP-2: histidine-rich protein-2; ITM: Institute of Tropical Medicine; PCR: polymerase chain reaction; pLDH: parasite lactate dehydrogenase; Pv-pLDH: *Plasmodium vivax*-specific parasite lactate dehydrogenase; RDT: rapid diagnostic test; WHO: World Health Organization.

## Competing interests

The authors declare that they have no competing interests.

## Authors' contributions

PG and JJ designed the study protocol. MvE and EB organized prospective sample collection. KB and PG carried out the test evaluations, LC performed PCR analysis and PG performed statistical analysis. PG, KB and JJ analysed and interpreted the results and drafted the manuscript. All authors contributed to the discussion of the results and the redaction of the manuscript, they all approved the final manuscript.
